# Lack of consideration of sex and gender in COVID-19 clinical studies

**DOI:** 10.1038/s41467-021-24265-8

**Published:** 2021-07-06

**Authors:** Emer Brady, Mathias Wullum Nielsen, Jens Peter Andersen, Sabine Oertelt-Prigione

**Affiliations:** 1 Danish Centre for Studies in Research and Research Policy, Aarhus University, Aarhus, Denmark; 2 Department of Sociology, University of Copenhagen, Copenhagen, Denmark; 3 Department of Primary and Community Care, Radboud University Medical Center, Nijmegen, Netherlands; 4 Medical Faculty OWL, University of Bielefeld, Bielefeld, Germany

**Keywords:** Health policy, Epidemiology, Clinical trials, Risk factors

## Abstract

Sex and gender differences impact the incidence of SARS-CoV-2 infection and COVID-19 mortality. Furthermore, sex differences influence the frequency and severity of pharmacological side effects. A large number of clinical trials to develop new therapeutic approaches and vaccines for COVID-19 are ongoing. We investigated the inclusion of sex and/or gender in COVID-19 studies on ClinicalTrials.gov, collecting data for the period January 1, 2020 to January 26, 2021. Here, we show that of the 4,420 registered SARS-CoV-2/COVID-19 studies, 935 (21.2%) address sex/gender solely in the context of recruitment, 237 (5.4%) plan sex-matched or representative samples or emphasized sex/gender reporting, and only 178 (4%) explicitly report a plan to include sex/gender as an analytical variable. Just eight (17.8%) of the 45 COVID-19 related clinical trials published in scientific journals until December 15, 2020 report sex-disaggregated results or subgroup analyses.

## Abstract (web summary)

Sex and gender have been associated with differences in SARS-CoV-2 incidence and clinical outcomes and therefore warrant consideration in study designs. Here, the authors assess registered and published clinical COVID-19 studies and find that sex-disaggregated analyses are infrequently presented or planned.

## Introduction

Available data point towards an increased risk of mortality for male patients with COVID-19 worldwide compared to female patients^1^. This could be related to intrinsic sex differences in the immune reaction^2^ or specific characteristics of the SARS-CoV-2 infectious process. The virus connects to the ACE2 receptor, which is encoded on the X chromosome and co-engages a serine protease – TMPRSS2 – that appears to be hormone-sensitive^3^. Reports also highlight the role of the innate immune response in the fight against the virus; specifically of TLR-7^4^, which is also encoded on the X chromosome. TLR-7 has been previously described as a relevant modulator of sex-specific differences in anti-viral immunity^5^. The investigation of sex differences could provide essential insights into COVID-19 pathophysiology and possibly aid the identification of effective interventions. In addition to the study of sex differences, gender-sensitive analysis is warranted^6^. Gender, a multidimensional variable describing identity, norms and relations between individuals^7^, can influence access to testing, diagnosis, medical care, and pharmacological treatments, and significantly affect the availability of social, economic, and logistic support^6^. Gender can also influence preventative and risk behavior, possibly impacting the course of the infection^8^. Both sex and gender can influence the pharmacokinetics, pharmacodynamics, and the safety profile of drugs^9,10^.

Several calls urging the inclusion of sex and gender into COVID-19 trials have been published^6,11,12^. Excluding one sex from clinical trials and not disaggregating results by sex can lead to an increased incidence of unwanted side effects in the untested population^13^ due to overmedication and other factors^14^. Not addressing the gender dimension hampers the opportunity to reduce inequality in healthcare, promote preventative action and modulate the course of the infection and pharmacological access^15^. Given these potential health risks for a large fraction of the infected population, we investigated the consideration of sex and/or gender as an analytical variable in currently registered and published trials for SARS-CoV-2/COVID-19.

In this work we show that attention to sex and gender at the registration and publication stages for COVID-19 studies is generally low. Only 178 (4%) of the 4420 studies in our ClinicalTrials.gov sample mention a plan to include sex/gender as an analytical variable, a further 237 (5.4%) plan sex-matched or representative samples or emphasized sex/gender reporting and 935 (21.2%) only mention sex/gender in the context of a recruiting statement. The majority of the sample (2946 studies, 66.7%) makes no mention of sex/gender in the study registration. Of the 45 publications for randomized control trials (RCTs) of pharmacological interventions for COVID-19 we identified, eight (17.8%) report sex-disaggregated results or subgroup analyses.

## Results

### Sex and gender in clinical study registrations

**Fig. 1:**
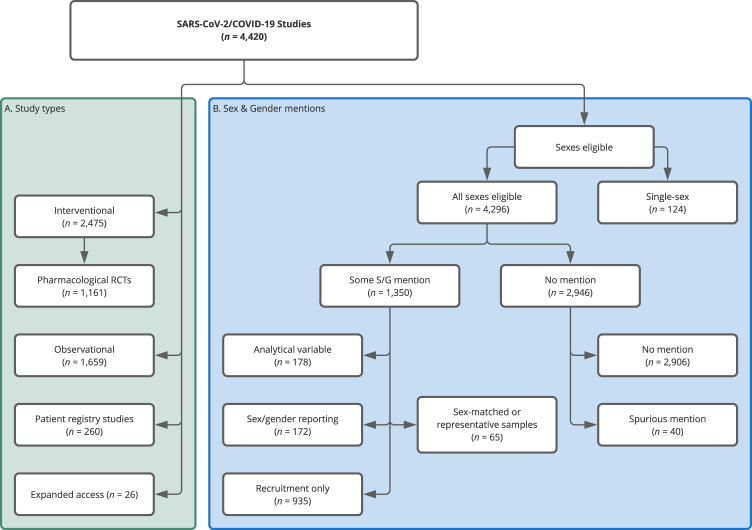
Study types and allocation into sex/gender (S/G) attention groups for the COVID-19 sample. The left-hand (green) panel shows the distribution of the 4420 identified studies across study types. We created the (Pharmacological) RCT label following the procedure outlined in Methods section, the other classifications are taken from ClinicalTrials.gov. The right-hand panel (blue) shows the distribution of studies across the various mutually exclusive sex/gender groups we defined (see Methods section). In the ‘Some S/G mention’ set there is a hierarchy. The most extensive consideration was sex/gender as an analytical variable and after that sex/gender matching/representation, followed by explicit statements of intent to record or report participant sex/gender. Finally, the ‘Recruitment Only’ contains those studies whose only sex/gender mention was in a recruiting context. We allocated the studies to one of the categories based on the highest category of S/G consideration. For instance, if they reported attention to S/G in analysis and recruiting, we only counted them in analysis.

We identify 4420 registered SARS-CoV-2/COVID-19 studies, of which 1659 are observational and 2475 interventional. Of the interventional trials, 1161 are randomized controlled trials (RCTs) of pharmacological (drug and biological/vaccine) interventions. In addition, we identify 260 patient registry studies and 26 expanded access/compassionate use studies (Fig. 1, for further specifications see Methods section).

Of the 4420 registered studies, 935 studies (21.2%) explicitly address sex/gender solely as a recruitment criterion, and only 178 studies (4%) mention sex/gender as a planned analytical variable. A further 237 (5.4%) plan sex-matched or representative samples (65) or emphasize sex/gender reporting (172). 124 studies (2.8%) focus solely on one sex, with 100 recruiting only female participants and 24 only male ones. Female-only studies mostly focus on the relation between COVID-19 and pregnancy outcomes. One study explicitly addresses the impact of COVID-19 on the transgender population. As for the remainder of the studies, 2906 (65.7%) do not address sex/gender in the study protocol registration on ClinicalTrials.gov beyond the mandated selection from a list of the eligible groups for the study (‘Male’, ‘Female’, or ‘All’), and 40 have a spurious mention unrelated to recruitment or analytical decisions. Of the 4420 studies, only 346 (7.8%) explicitly mention “gender” (in any context) in the sections of the study protocol targeted by our search (see Methods section). The level of detail in the study records is very variable. Only 70.1% (3100) include any information in the detailed description field and only 132 attach supporting pdfs (79 included statistical analysis plans). The median total word count across the two study description fields (brief and detailed) is 245 for the observational studies, 260 for the other interventions, and 214 for pharmacological RCTs.

**Fig. 2:**
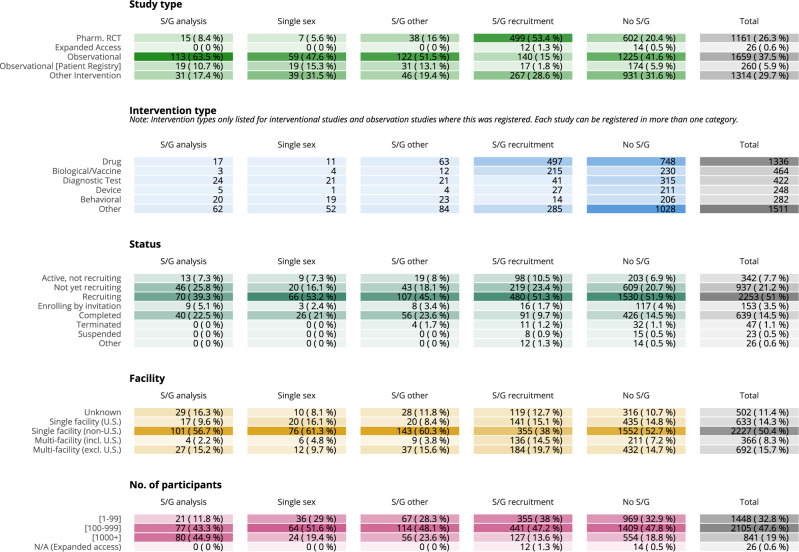
Properties of COVID-19 Studies in the sample and level of attention to sex/gender (S/G). Sex/gender coding and properties of studies at time of data collection on 26 January 2021. Studies are reported according to different analytical criteria. Color blocks represent one category of analysis. Studies are exclusively allocated to one category per analysis, with the exception of the “intervention” category. Here, studies can propose multiple interventions and, hence, appear under each unique intervention type they include. In the facility section, we focus on the number (single or multiple) and location (US or non-US) of the facilities recruiting participants into the studies. Address information for facilities is available for 89% of our full COVID-19 set. All studies (except the 26 Expanded Access) provide enrollment details and they are mostly ‘anticipated’, i.e. target numbers (80%).

As displayed in Fig. 2, attention to sex/gender in the study design and registration phase is generally low and varies by study type. The study protocols that mention sex/gender as an analytical variable are mostly observational and patient registry studies (132, 74.2%), as are those with sex/gender matching/reporting (153, 64.6%). Interventional protocols are dominated by studies that only discuss sex/gender in the context of recruitment and studies with no mention of sex/gender at all. Only 15 (1.3%) of the 1161 pharmacological intervention RCTs and 31 (2.4%) of the 1314 other interventional studies have registered a plan to consider sex as a variable upon analysis.

Studies intending to conduct sex-analyses are larger on average (median: 700 anticipated participants) than those with no sex mention at all (median: 176 anticipated participants), but again study type plays a role. Observational and patient registry studies enroll 287 and 400 patients on average, compared to 160 and 100 for pharmacological RCTs and other intervention trials. Phase information is provided for 1583 (64%) of the other intervention and pharmacological RCTs, and are dominated by phase 2 and 3 studies (70.1%, 1109 studies). The US is the most common host country of participating facilities; 1003 of the studies have at least one recruiting location in the US, followed by France (603), the United Kingdom (228), Italy (213), and Spain (211) (see Supplementary Fig. 2). Of studies with a recruiting facility based in the United States (US), 242 (24.2%) are observational and 2.1% (21) plan sex/gender analysis. Of those with no US presence, 1417 (41.4%) are observational and 4.6% (157) plan sex/gender analysis (see Fig. 2).

### Registration patterns during the pandemic

Our analysis shows that attention to sex/gender in trial registrations remained low throughout the pandemic. Monthly study registrations (as measured by date first submitted to ClinicalTrials.gov) peaked in late spring and early summer of 2020 (see Supplementary Fig. 1, panel B) and 56% (2475) of our sample was submitted in the first half of 2020. Of the 2475 studies submitted in the first half of 2020, 102 (4.1%) indicate a plan to include sex as an analytical variable, while 76 (3.9%) of the 1945 submitted in the second half indicate such a plan. The level of other mentions (sex-matching, sex-representative sample, explicit statement of intent to record, and report sex/gender etc.) also remained low throughout the year. Supplementary Figure 1, panel A, plots developments in the level of attention to sex and gender per month, from January 2020 to January 2021.

As of 26 January 2021, 639 registered studies are completed, and 18 of them have reported results on ClinicalTrials.gov. Of these, one registration is a female-only study. Of the remaining 17, three have indicated an intent to include sex/gender as an analytical variable upon registration, while none have uploaded sex-based analysis as part of their results.

### Sex and gender in trial publications

A PubMed search of published RCTs retrieved 957 results of which 56 (6%) are original reports of RCTs (for further specifications see Data and Methods section). Eleven are excluded due to a focus on non-pharmacological COVID-19-related interventions. Fourteen trials are phase III, the others phase I and II. All of the remaining 45 trials report the number of female and male participants. Of these, 4 (9%) adjust their analysis by sex/gender and 8 (17.8%) report sex-disaggregated analyses. These analyses are e.g. pre-specified subgroup analyses, sex-specific reporting of side effects or sex-stratified analyses.

## Discussion

Although sex appears to be an important determinant of mortality risk and immunologic responses to COVID-19^5,16,17^, currently registered clinical trials mostly omit sex as an explicit recruitment or analytical criterion. Of the identified RCT publications, only 17.8% report sex-specific analyses or disaggregate their results by sex. This suggests that only a small share of the studies lacking consideration of sex upon registration at ClinicalTrials.gov, account for sex during trial execution and reporting, leading to a significant information gap. Gender is given even less consideration. Very few studies address gender as a recruitment or analytical variable. This applies equally to observational and interventional trials.

As research into COVID-19 pathophysiology and pharmacological interventions has led to substantial knowledge gain, the impact of sex and gender differences on these factors has also become more evident. Research demonstrates the impact of sex on clinical presentation^18^, severity of disease^19^, and treatment allocation^20^. The incidence of side effects with hydroxychloroquine^21^ or anaphylaxis upon vaccination^22^ are both higher in female subjects. Furthermore, the recently reported sinus vein thromboses associated with two of the vector-based vaccines also appear to affect female subjects more often^23,24^. These reports confirm the previously described discrepancies in sex-specific pharmacological response^14^ and highlight the need for sex-disaggregated data collection, analysis, and reporting. However, despite increasing awareness, the level of attention to sex and gender in ClinicalTrials.gov registrations remained low and relatively stable from January 2020 to January 2021.

Several aspects need to be taken into consideration when looking at our findings. First, the size of trials might impact the inclusion of sex as an analytical variable. Interventional trials in the sample tended to be smaller, which might limit the ability of experimenters to disaggregate analyses while maintaining statistical power. The small sample sizes may partly be explained by experimenters facing challenges with participant enrollment^25^. However, given the striking sex and gender differences identified for this disease, a lack of consideration of these variables could undermine the reproducibility and generalizability of the results. Ignoring sex and gender aspects of COVID-19 is not scientifically or ethically justifiable, and interventional trials should be designed to account for these variables. Second, disciplinary culture might affect the decision to perform sex and gender-sensitive analysis. For example, researchers conducting large observational studies might be trained to consider a vast array of social determinants, which biomedical researchers might not prioritize in interventional trials. Our results reflect these differences, as observational studies appeared to mention sex and gender more frequently in their registration files. In addition to sex and gender, this type of research highlights the impact of a wide range of social determinants on the development of COVID-19, including poverty^26^, racialized disparities^27^, and the impact of social context^28^. Biomedical research into the viral dynamics in people cannot ignore these determinants. Third, national requirements for trial performance might play a role in the decision to include sex and/or gender as analytical variables. We chose to examine the ClinicalTrials.gov database because of the quality and detail of its registrations and because the US has clear guidance on the need for consideration of sex (and gender) in clinical studies^29^. However, by focusing on ClinicalTrials.gov, we will have missed a large proportion of studies conducted outside of the US, which limits the global validity of our findings.

Finally, researchers may not be providing sufficient detail on their planned analyses upon study registrations for us to detect the true level of attention they afford sex and gender. However, given that only 17.8% of the published trials reported sex-disaggregated analyses, a lack of detail upon registration does not appear to be substantially corrected during the execution, analysis, and publication process for the vast majority of trials. An enforced requirement of analytical details and quality control upon registration could possibly increase the consideration of sex and gender in clinical trials and improve the reliability of the published results.

In light of the current results, we urge researchers working in the field of SARS-CoV2 and COVID-19 to systematically apply a sex-specific methodology. This entails: (a) the recording of sex of all participants; (b) the inclusion of sex as an independent variable into multivariate analysis; (c) the performance of sex-disaggregated analyses; and (d) the reporting of sex-disaggregated results for unambiguous identification of differences in effectiveness, side effects, and mortality^30^. In addition, data for trials that record the sex/gender of participants, but are not statistically powered to disaggregate results by sex, should be made publicly available for pooling in evidence synthesis. More comprehensive analyses should address gender-related impacts^31^ and utilize intersectional approaches to identify specific subgroup experiences and barriers to access^32^.

The current experiences should inform future preparedness efforts. A general sex- and gender-sensitive approach should be structurally implemented through mandatory reporting requirements upon registration of clinical trials. This policy should be institutionalized to guarantee its application also in times of crisis. The striking sex differences in COVID-19 mortality highlight a need for universal consideration of these variables. Sex-specific analysis cannot be an afterthought once the acute phase has passed; it is proving to be an important contributor to the dissection of disease-specific mechanisms^17^. Investigating sex differences can highlight otherwise ignored mechanisms and should, hence, be an essential component of robust, reproducible, and socially relevant research.

## Methods

Data was obtained through a query of the relational database “Aggregate Analysis of ClincalTrials.gov” (AACT)^33^ on 26 January 2021. AACT contains all publicly available ClinicalTrials.gov data.

### Selection of the COVID-19 studies

In our search for COVID-related studies, we targeted the following trial-registration fields: the official and brief trial titles, the brief summary, the detailed description and conditions, as well as the titles and descriptions of outcome measures (primary, secondary, and other). The search terms used were: Coronavirus, Corona Virus, SARS-CoV-2, SARS CoV2, SARSCoV2, COVID, 2019 nCov, and 2019nCoV. The search was case-insensitive and blind to use of hyphens. Ninety eight percent of the protocols in our final sample had one of these search terms in their titles or conditions. The remaining 2% included studies, where COVID-19 or its synonyms were mentioned in the primary outcome measures or appeared in at least two other relevant fields, e.g. in a description of a non-primary outcome measure AND in the brief summary or detailed description. We adopted this approach to avoid including study registrations that mention COVID-19 but do not focus on health aspects directly related to the virus. Our approach was based on what we learned in an earlier round of analysis, where we manually inspected all studies without a COVID-19 term in the titles or conditions.

Our analysis covered registrations with a starting or submission date (if no starting date was available) between 1 January 2020 and 26 January 2021. We removed duplicate registrations and studies listed as ‘Withdrawn’ or ‘No longer available’, leaving us with a final sample of 4420 studies.

### Identification of attention to sex and gender

ClinicalTrials.gov mandates researchers to state the sexes eligible for their study by selecting one option from a pre-defined list (‘All’, ‘Male’ or ‘Female’). We used this data element to identify single-sex study designs. For registrations open to ‘All’ sexes, we identified attention to sex/gender by searching for the following terms (and their plurals): sex, gender, woman, female, man, male, girl, boy, pregnan*, and transg*.

We restricted these searches to the following registration fields: titles, brief summary, detailed description, eligibilities (population, gender, and inclusion criteria), study design group descriptions and titles, intervention descriptions, and primary, secondary, and other outcomes.

Two independent coders (EB and SOP) went through all registrations mentioning sex or gender-related terms, assigning each registration to one of the following categories: (a) sex/gender as an analysis criterion; (b) other sex/gender mentions (c) sex/gender recruitment; and (d) no relevant sex/gender mention. As of 26 January 2021, 79 studies had uploaded supporting pdf documents with indications that they included a statistical analysis plan and 76 of these were open to all sexes. We performed the same sex term search and coding on these 76 documents.

For inclusion into category (a) (analysis criterion), we looked for evidence of intention to include sex/gender as an analytical variable. For instance, if a registration stated that results would be stratified by sex, sex-subgroup analyses would be performed, sex would be included as a covariate, or that researchers hypothesized that sex/gender would affect outcomes, we considered the study permissible, and no further statistical details were expected. If sex/gender representation and differences were addressed in an introduction or literature summary but no mention was made in the outline of the data analysis protocol, we did not consider the protocol eligible for inclusion in category (a). Similarly, if ‘demographic variables’ were included in the outline of the planned statistical analysis, but sex/gender was not explicitly mentioned in that particular discussion, we did not include that study. We did not consider the intention to sex-match the treatment and control groups a strong enough signal of planned sex/gender analysis, and coded registrations including such intentions separately in category b).

We assigned protocols to category (b) (other sex/gender mentions) if they explicitly stated that they would record or report the sex of the participants or would sex match to controls, aim for a representative sample etc. None of these protocols clearly indicated any intention to include the variable upon analysis.

Category (c) (sex/gender recruitment) consists of protocols that only addressed sex/gender in the context of an explicit recruitment statement covering both sexes/genders. The sole selection of “All” in the pre-defined sexes eligible for study field was not considered sufficient, as this registration step does not represent a marker of a specific focus on the topic. Likewise, a focus on the sex of donors or parents, but not the recipients or children, who were the focus of the study, was not considered sufficient for inclusion. Category (c) includes a dominant subset of studies that were included automatically, i.e., without manual coding (777 of 935) owing to their large number and the type of sex-term match. These were studies that only mentioned sex/gender terms in the list of eligibility inclusion criteria and nowhere else in the registration, implying they could only be candidates for sex/gender recruitment. We caution that this subset may include some noise (e.g. studies that specify the type of contraception that both sexes would be required to use to be eligible for inclusion, while not having a separate explicit recruitment statement). However, this possible bias would lead to an overestimation rather than an underestimation for category (c).

Studies in category (d) (no sex/gender mention) either had no match to one of our sex/gender search terms (the vast majority), or were spurious sex matches. The latter set includes studies where, for example, the only mention of sex was in a literature summary or in the context of contraception requirements and pregnancy tests.

### Identification of the pharmacological RCTs

Our COVID-19 sample contains many studies that investigate the social impact of COVID-19 or the effect of the pandemic on access to healthcare and treatment. These are mainly concentrated amongst the observational studies. We created a separate ‘pharmacological RCT’ category, consisting of the drug and vaccine/biological interventional RCTs, to have a sample of the most relevant and high-quality trials. We retained the rest of the interventional trials (now labeled ‘Other Intervention’) in addition to the observational and patient registry registrations as they do contain research of disease prevalence, progression, and wider effects on the population. ClinicalTrials.gov does not have a pre-defined pharmacological RCT label. To create one, we made use of several data elements, where ClinicalTrials.gov requires those registering interventional studies to select an option from a pre-defined list. These data elements were study type, allocation into study groups, intervention type(s), and study group type. We selected interventional studies with randomized allocation into study groups, having least one drug or vaccine/biological intervention and a participant group of type ‘placebo/sham comparator’ or ‘no intervention’. Since the ‘experimental’ and ‘active comparator’ group-type labels were attached to some groups that were obvious controls, we also proceeded to search the group titles and descriptions for occurrences of control and placebo. We collected a few more studies by looking at the study titles and the descriptions of the interventions for occurrences of *RCT* or random* + control*/placebo*. Our final pharmacological intervention RCT set has 1161 studies. All remaining interventional trials are labeled ‘Other Intervention’.

### Selection and coding of the COVID-19 publications

We searched the PubMed database on 15 December 2020 for publications on SARS-CoV-2/COVID-19 trials using the search strategy for COVID-19 provided by the Canadian Agency for Drugs and Technologies in Health (CADTH)^34^ and the RCT search filter of the Scottish Intercollegiate Guideline Network (SIGN)^35^, resulting in 957 unique article records. Two independent coders (MWN and SOP) screened these 957 articles to identify original publications of randomized controlled trials. We excluded comments, reviews, observational studies, trial protocols, post-hoc analyses, non-randomized trials, and RCTs not focusing on COVID-19.

Two independent coders (MWN and SOP) evaluated the degree of sex/gender-specific analysis in the 56 original reports of RCTs. Sex/gender-specific analysis could range from (a) sole mention of participating numbers of women/men in the trial, (b) explicit incorporation of sex/gender as analytical variable, and (c) reporting of sex/gender-disaggregated analyses.

### Reporting summary

Further information on research design is available in the Nature Research Reporting Summary linked to this article.

## Supplementary information

Supplementary Information

NR_Reporting Summary

## Data Availability

Source data for this study was gathered from the following public repositories; ClinicalTrials.gov study registration data from the Aggregate Analysis of ClincalTrials.gov database (https://aact.ctti-clinicaltrials.org/) and publications from the PubMed database of biomedical literature (https://pubmed.ncbi.nlm.nih.gov/). Data on our final study and paper samples, which supports our main results, is available in an Excel file in a public Github repository at https://github.com/bradyemer/Sex-gender-in-COVID-19-trials (10.5281/zenodo.4772598)

## Code availability

The AACT database of ClinicalTrials.gov data was queried using R (v. 3.6.1). Python (v. 3.8.5) was used to clean the data, create the COVID-19 and sex/gender candidate samples, the final numbers for this paper and the supplementary figures. This work was documented in a Jupyter (v. 4.6.3) Python 3 notebook. Manual classification of study registrations with sex/gender terms and of the PubMed sample was documented in Excel (v. 16.33). Figures 1 and 2 were created in R (v. 4.0.2). An R and Python-based pipeline which reproduces our selection of the ClinicalTrials.gov COVID-19 sample and sex/gender candidates is publicly available in a Github repository at https://github.com/bradyemer/Sex-gender-in-COVID-19-trials (10.5281/zenodo.4772598)
