# Supplementary material for: Lack of consideration of sex and gender in COVID-19 clinical studies
Source: Nat Commun. 2021 Jul 6;12:4015. doi: 10.1038/s41467-021-24265-8 (PMC8260641; doi:10.1038/s41467-021-24265-8)
Supplement: Supplementary file 1 — Supplementary Information [file 41467_2021_24265_MOESM1_ESM.pdf]

Supplementary Information for Lack of Consideration of Sex and Gender in COVID-19 Clinical Studies

Emer Brady[1], Mathias Wullum Nielsen[2], Jens Peter Andersen[1], Sabine Oertelt-Prigione[3,4]

[1]Danish Centre for Studies on Research and Research Policy, Aarhus University, Aarhus, Denmark
[2]Department of Sociology, University of Copenhagen, Copenhagen, Denmark
[3]Department of Primary and Community Care, Radboud University Medical Center, Nijmegen, Netherlands
[4]Medical Faculty OWL, University of Bielefeld, Bielefeld, Germany

Contents

Supplementary Figures

Supplementary Figure 1: Level of attention to sex/gender per month……………….2
Supplementary Figure 2: Number of studies taking place in the top-20 countries hosting recruiting facilities………………………………………………………….3

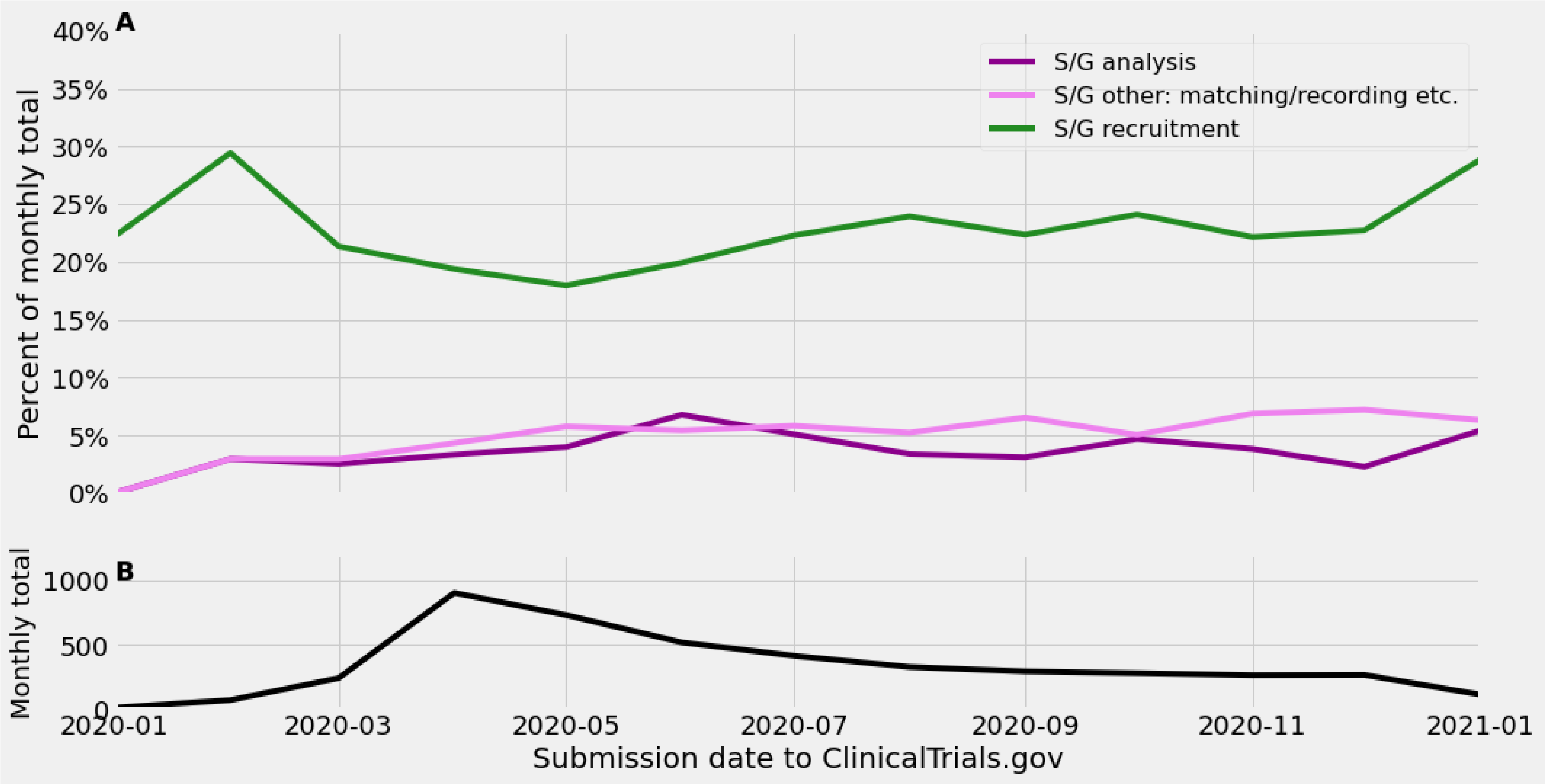

Supplementary Figure 1. Level of attention to sex/gender per month. Panel A: For all studies first submitted to ClinicalTrials.gov in a given month, we show the fraction that fall into the three main sex/gender categories we coded for (not shown are single sex and no sex/gender studies). Note that study registrations can be updated at any time after submission and that we coded the studies into sex/gender categories using the information available in the registrations on ClinicalTrials.gov in January 2021. Panel B shows the monthly counts of studies submitted to ClinicalTrials.gov for our full set of 4420. We used the 'study first submitted date' on the shared x-axis, since all studies have one and it is generally required that they are registered before they start, though this is not always adhered to.

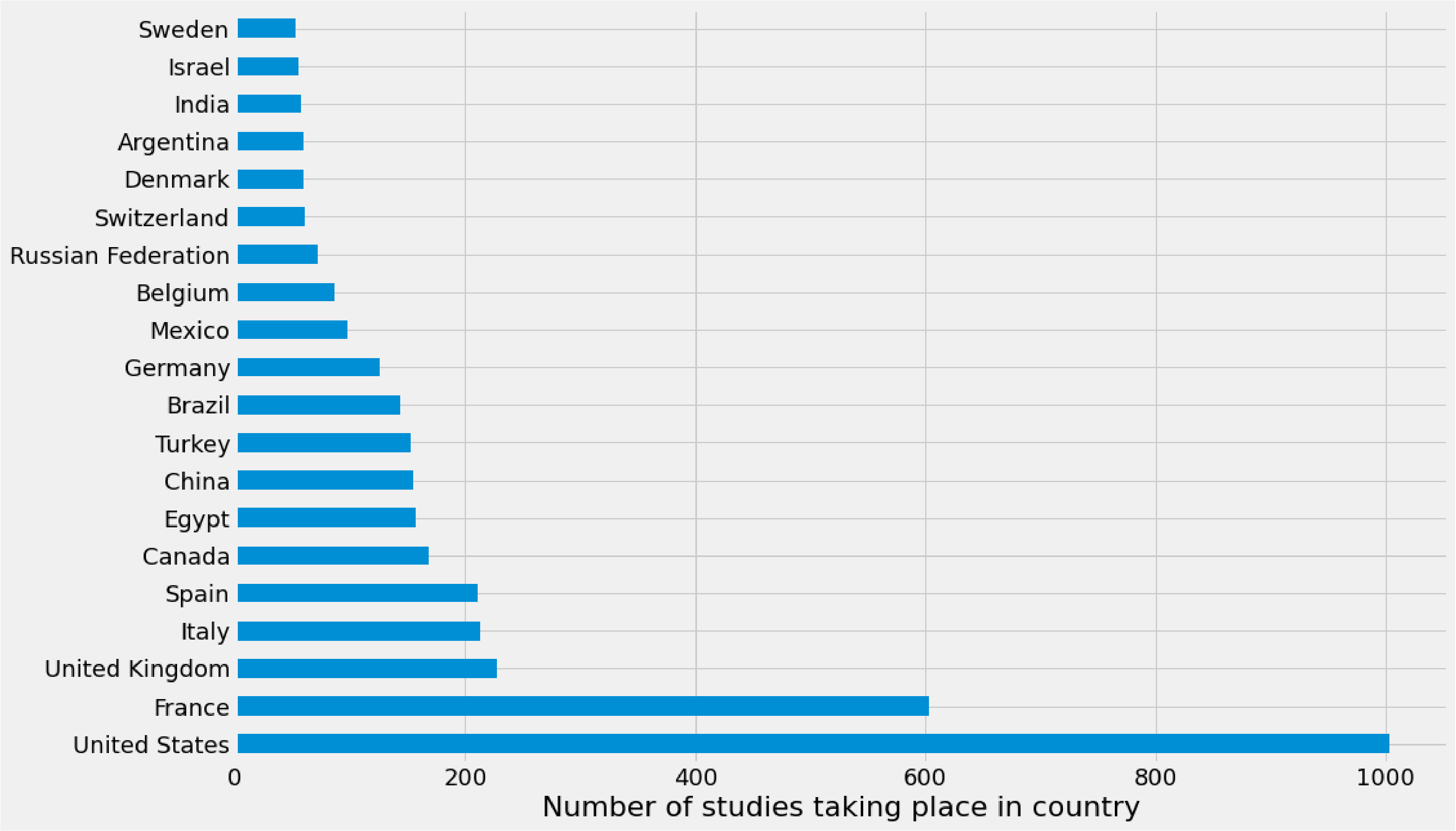

Supplementary Figure 2. Number of studies taking place in the top-20 countries hosting recruiting facilities. A study is counted once under each country that hosts one or more of its recruiting facilities. 3920 studies provided addresses with country information for their participating facilities**.**
