# Supplementary material for: Lack of consideration of sex and gender in COVID-19 clinical studies
Source: Nat Commun. 2021 Jul 6;12:4015. doi: 10.1038/s41467-021-24265-8 (PMC8260641; doi:10.1038/s41467-021-24265-8)
Supplement: Supplementary file 2 — NR_Reporting Summary [file 41467_2021_24265_MOESM2_ESM.pdf]

nature research

Corresponding author(s): Sabine Oertelt-Prigione
Last updated by author(s): May 19, 2021

Reporting Summary

Nature Research wishes to improve the reproducibility of the work that we publish. This form provides structure for consistency and transparency in reporting. For further information on Nature Research policies, see our Editorial Policies and the Editorial Policy Checklist.

Statistics

For all statistical analyses, confirm that the following items are present in the figure legend, table legend, main text, or Methods section.

n/a	Confirmed	
☐	☒	The exact sample size (n) for each experimental group/condition, given as a discrete number and unit of measurement
☒	☐	A statement on whether measurements were taken from distinct samples or whether the same sample was measured repeatedly
☒	☐	The statistical test(s) used AND whether they are one- or two-sided<br>*Only common tests should be described solely by name; describe more complex techniques in the Methods section.*
☒	☐	A description of all covariates tested
☒	☐	A description of any assumptions or corrections, such as tests of normality and adjustment for multiple comparisons
☒	☐	A full description of the statistical parameters including central tendency (e.g. means) or other basic estimates (e.g. regression coefficient) AND variation (e.g. standard deviation) or associated estimates of uncertainty (e.g. confidence intervals)
☒	☐	For null hypothesis testing, the test statistic (e.g. F, t, r) with confidence intervals, effect sizes, degrees of freedom and P value noted<br>*Give P values as exact values whenever suitable.*
☒	☐	For Bayesian analysis, information on the choice of priors and Markov chain Monte Carlo settings
☒	☐	For hierarchical and complex designs, identification of the appropriate level for tests and full reporting of outcomes
☒	☐	Estimates of effect sizes (e.g. Cohen's d, Pearson's r), indicating how they were calculated

Our web collection on statistics for biologists contains articles on many of the points above.

Software and code

Policy information about availability of computer code

Data collection	R (v. 3.6.1) was used to connect to and query the cloud version of the PostgreSQL AACT database (of all studies registered on ClinicalTrials.gov) on Jan 26, 2021. All available data for studies registered or starting between Jan 1, 2020 and Jan 26th 2021 was downloaded.
Data analysis	Work (code and output) was documented in a Jupyter (v. 4.6.3) Python 3 notebook by E.B.<br>Python (v. 3.8.5) was used by E.B. to clean the downloaded data and perform text searches to create the COVID-19 dataset and select the sex/gender candidate studies for manual review.<br>A list of the studies with sex/gender mentions was exported to Excel (v. 16.33) and the results of the manual classification were documented in that file by E.B and S.O.P.<br>Manual classification of the Pubmed publication sample was performed by S.O.P and M.W.N and documented in Excel.<br>Final numbers and supplementary figures were produced in Python by E.B and Figures 1 and 2 were created in R (v. 4.0.2) by J.P.A.<br>Examples of the R queries and of the Python code used to generate the COVID-19 sample as well as an Excel file of the final sample of 4420 studies and their sex/gender labels is available in a public Github repository at https://github.com/bradyemer/Sex-gender-in-COVID-19-trials (doi: https://doi.org/10.5281/zenodo.4772598)

For manuscripts utilizing custom algorithms or software that are central to the research but not yet described in published literature, software must be made available to editors and reviewers. We strongly encourage code deposition in a community repository (e.g. GitHub). See the Nature Research guidelines for submitting code & software for further information.

Data

Policy information about availability of data

All manuscripts must include a data availability statement. This statement should provide the following information, where applicable:

- Accession codes, unique identifiers, or web links for publicly available datasets
- A list of figures that have associated raw data
- A description of any restrictions on data availability

Source data for this study was gathered from public repositories; ClinicalTrials.gov study registration data from the Aggregate Analysis of ClincalTrials.gov (AACT) database (https://aact.ctti-clinicaltrials.org/) and publications from the PubMed database of biomedical literature (https://pubmed.ncbi.nlm.nih.gov/). Data on our final study and paper samples, which supports our main results, is available in a public Github repository at https://github.com/bradyemer/Sex-gender-in-COVID-19-trials (doi: https://doi.org/10.5281/zenodo.4772598)

Field-specific reporting

Please select the one below that is the best fit for your research. If you are not sure, read the appropriate sections before making your selection.

☐ Life sciences ☒ Behavioural & social sciences ☐ Ecological, evolutionary & environmental sciences

For a reference copy of the document with all sections, see nature.com/documents/nr-reporting-summary-flat.pdf

Behavioural & social sciences study design

All studies must disclose on these points even when the disclosure is negative.

Study description	Quantitative study investigating the attention to sex and gender in COVID-19 clinical trials registered on ClinicalTrials.gov or published on PubMed
Research sample	The clinical study sample was drawn from the ClinicalTrials.gov repository, which has been converted into a publicly available relational database (AACT, updated daily) by the Clinical Trials Transformation Initiative.<br>The sample consists of all COVID-19 trials registered on ClinicalTrials.gov between Jan 1, 2020 and Jan 26 2021, it should be broadly representative of all COVID-19 research, though likely with a US bias.<br>The sample of published trials consists of peer-reviewed articles listed on the PubMed repository reporting the results of Drug RCT trials on COVID-19 published on/before December 15, 2020.
Sampling strategy	The trial titles, conditions, brief summaries, detailed descriptions and outcomes measures were searched for COVID-19 related terms (this was an iterative process where new terms were added as appropriate). All trials with a keyword match in the title or conditions were accepted as a COVID-19 trial without further review (98% of final sample). Studies with a COVID-19 term in a primary outcome were also included. After that we required studies to have a COVID-19 term in the non-primary outcomes AND the brief summary or in the brief summary AND detailed description to ensure COVID-19 was a main focus of the study.<br>Candidates for studies considering sex/gender as an analytical variable, other sex/gender mentions (matching/reporting/representation) or considering sex/gender upon recruitment were selected by a text search (from a list of predetermined keywords related to sex and gender) of the titles, brief summaries, detailed descriptions, eligibility criteria, outcomes measures, design groups and interventions data fields of those studies that indicated all sexes were eligible for participation. We also identified the coded the statistical analysis plans (in pdf form) that 76 studies (open to all sexes) had provided and manually coded these too.<br><br>To identify the Pubmed data we searched the PubMed database on December 15, 2020 for publications on SARS-CoV-2/COVID-19 trials using the search strategy for COVID-19 provided by the Canadian Agency for Drugs and Technologies in Health (CADTH) [https://covid.cadth.ca/literature-searching-tools/cadth-covid-19-search-strings/#covid-19-medline] and the RCT search filter of the Scottish Intercollegiate Guideline Network (SIGN) [https://www.sign.ac.uk/assets/search-filters-randomised-controlled-trials.docx], resulting in 957 unique article records.
Data collection	Data were collected from two public repositories; the AACT relational database of ClinicalTrials.gov clinical study registrations and the PubMed database of biomedical literature. E.B collected all available data from the AACT database for study registrations uploaded to ClinicalTrials.gov, or starting, during the period Jan 1, 2020 to Jan 26, 2021. This source data was collected using R and saved as csv files. J.P.A searched PubMed for papers reporting results of COVID-19 randomized control trials using the CADTH COVID-19 search strategy and the SIGN RCT search filter. The results were saved in a Excel file. Both E.B and J.P.A were not blind to the study hypothesis during data collection.
Timing	Trial data were initially gathered in a Dec 4 2020 query of the AACT cloud database, updated again on Jan 15 2021 (before coding for sex/gender) and finally in a Jan 26th 2021 query. Pubmed data were collected on Dec 15, 2020.
Data exclusions	5 obvious duplicates, 82 'Withdrawn studies and 5 'No Longer available' were removed from our COVID-19 sample. Only studies starting on/after Jan 1 2020 or, if no start date available, first submitted to ClinicalTrials.gov on/after Jan 1 2020 were included in our sample.<br>901 publications that were not reports of randomized control trials and a further 11 RCT papers that did not focus on pharmacological interventions were excluded from the paper sample.
Non-participation	None

Randomization	No participants were allocated into experimental groups

Reporting for specific materials, systems and methods

We require information from authors about some types of materials, experimental systems and methods used in many studies. Here, indicate whether each material, system or method listed is relevant to your study. If you are not sure if a list item applies to your research, read the appropriate section before selecting a response.

Materials & experimental systems

n/a	Involved in the study
☒	☐ Antibodies
☒	☐ Eukaryotic cell lines
☒	☐ Palaeontology and archaeology
☒	☐ Animals and other organisms
☒	☐ Human research participants
☒	☐ Clinical data
☒	☐ Dual use research of concern

Methods

n/a	Involved in the study
☒	☐ ChIP-seq
☒	☐ Flow cytometry
☒	☐ MRI-based neuroimaging
